# A Transitional Care Model for Veterans With Complex Needs During COVID: The Behavioral Recovery Outreach (BRO) Team

**DOI:** 10.1093/geroni/igab046.172

**Published:** 2021-12-17

**Authors:** Kathleen Matthews, Latrice Vinson

## Abstract

The Veterans Health Administration’s Care for Patients with Complex Problems (CP)2 Program developed a national infrastructure to disseminate promising practice models to improve care for Veterans with complex medical, mental health, and/or neurocognitive conditions, who may also have behaviors disruptive to care. A strategic priority is improving safe and effective transitions to community care for Veterans with complex care needs, many of whom have historically been limited to VA settings as a result of behavioral concerns. The Behavioral Recovery Outreach (BRO) Team was the first model identified for national dissemination and evaluation at partner sites. Developed at VA Central Iowa, BRO is an interdisciplinary team model that identifies Veterans in long-term VA care settings with complex care needs to engage in individualized behavioral programing to manage/stabilize behaviors and safely transition them to more appropriate and less costly community settings. This symposium will describe the BRO team model, highlight the facilitators and barriers to nationally disseminating the BRO model with VA partner facilities, discuss adaptations in continuing community transitions following the COVID-19 pandemic, and describe program outcomes. The first speaker will discuss development of the BRO model and outcomes of a regional dissemination. The second speaker will present results from the program evaluation of the national dissemination. The final speaker will describe BRO Team expansion and lessons learned from the perspective of a VA partner facility. The (CP)2 Program Director will integrate findings and highlight implications for scaling and evaluating promising models for national dissemination for policy, practice, and future research.

